# Occurrence of Extended Spectrum β-Lactamases, KPC-Type, and MCR-1.2-Producing *Enterobacteriaceae* from Wells, River Water, and Wastewater Treatment Plants in Oltrepò Pavese Area, Northern Italy

**DOI:** 10.3389/fmicb.2017.02232

**Published:** 2017-11-10

**Authors:** Mariasofia Caltagirone, Elisabetta Nucleo, Melissa Spalla, Francesca Zara, Federica Novazzi, Vittoria M. Marchetti, Aurora Piazza, Ibrahim Bitar, Marica De Cicco, Stefania Paolucci, Giorgio Pilla, Roberta Migliavacca, Laura Pagani

**Affiliations:** ^1^Unit of Microbiology and Clinical Microbiology, Department of Clinical-Surgical, Diagnostic and Pediatric Sciences, University of Pavia, Pavia, Italy; ^2^Department of Biomedical and Clinical Sciences, Romeo and Enrica Invernizzi Pediatric Research Center, University of Milan, Milan, Italy; ^3^Faculty of Medicine, Charles University, Plzen, Czechia; ^4^Molecular Virology Unit, Microbiology and Virology Department, Fondazione IRCCS Policlinico San Matteo, Pavia, Italy; ^5^Department of Earth and Environment Sciences, University of Pavia, Pavia, Italy

**Keywords:** water ecosystems, Gram-negative bacteria, carbapenemases, colistin resistance, molecular characterization

## Abstract

To evaluate the water compartment antibiotic-resistance contamination rates, 11 wells, five streams, and four treatment plants located in the Oltrepò Pavese area were screened for the presence of third generation cephalosporins resistant Gram-negative bacteria. *Enterobacteriaceae* were also characterized for the Extended-Spectrum-β-Lactamases (ESBLs), carbapenemases, and *mcr-1* genes presence. From December 2014 to November 2015, 246 water samples were filtered, plated on Plate Count Agar, MacConkey Agar, and MacConkey Agar with cefotaxime. Isolates were species identified using AutoSCAN-4-System and ESBLs, carbapenemases, and colistin resistance determinants were characterized by PCR, sequencing, and microarray. Plasmid conjugative transfer experiments, PCR-based Replicon typing, Pulsed-Field Gel Electrophoresis, Multi-Locus-Sequence-Typing, and *in-silico* plasmid characterization were performed. A total of 132 enterobacteria isolates grew on MacConkey agar with cefotaxime: 82 (62.1%) were obtained from streams, 41 (31.1%) from treatment plants, and 9 (6.8%) from wells. Thirty out of 132 (22.7%) isolates, mainly belonging to *Escherichia coli* (*n* = 15) species, showed a synergic effect with piperacillin-tazobactam. A single ESBL gene of *bla*_CTX−M_-type was identified in 19/30 isolates. In further two *E. coli* strains, a *bla*_CTX−M−1_ gene co-existed with a *bla*_SHV_-type ESBL determinant. A *bla*_SHV−12_ gene was detected in two isolates of *E. coli* (*n* = 1) and *Klebsiella oxytoca* (*n* = 1), while any ESBL determinant was ascertained in seven *Yersinia enterocolitica* strains. A *bla*_DHA_-type gene was detected in a cefoxitin resistant *Y. enterocolitica* from a stream. Interestingly, two *Klebsiella pneumoniae* strains of ST307 and ST258, collected from a well and a wastewater treatment plant, resulted KPC-2, and KPC-3 producers, respectively. Moreover, we report the first detection of *mcr-1.2* ST10 *E. coli* on a conjugative IncX4 plasmid (33.303 bp in size) from a stream of Oltrepò Pavese (Northern Italy). Both ESBLs *E. coli* and ESBLs/carbapenemase-producing *K. pneumoniae* strains showed clonal heterogeneity by Pulsed-Field Gel Electrophoresis and Multi-Locus-Sequence-Typing. During one-year study and taking in account the whole Gram-negative bacterial population, an average percentage of cefotaxime resistance of 69, 32, and 10.3% has been obtained for the wastewater treatment plants, streams, and wells, respectively. These results, of concern for public health, highlight the need to improve hygienic measures to reduce the load of discharged bacteria with emerging resistance mechanisms.

## Introduction

Antibiotic resistance, in particular to third generation cephalosporins (3GCs) and carbapenems, threatens healthcare globally. Drug resistance has traditionally been viewed as a clinical problem, but recently natural ecosystems have been recognized as an important reservoir of antibiotic resistance genes (ARGs) (Berglund, 2015).

In aquatic environments the prevalence of antibiotic-resistant bacteria, which may originate from anthropogenic sources such as hospital and municipal effluents, is constantly growing (Baquero et al., 2008; Bouki et al., 2013). The large amounts of antibiotics or their active metabolites released into wastewater with treated urine and feces increase the selective pressure in bacterial populations, allowing the development of antibiotic-resistant microbes' generations (Davies and Davies, 2010).

Furthermore, the surface water could act as resistance hotspots where ARGs disseminate favored by bacteriophages or integrons and new resistant strains are created by horizontal gene transfer (Berglund, 2015; Colombo et al., 2017).

β-lactamase genes have been identified in bacteria isolated from both surface waters and Wastewater Treatment Plants (WWTPs) (Schwartz et al., 2003; Poppe et al., 2006; Bouki et al., 2013). The β-lactamases now include >2,000 naturally occurring amino acid sequences. Some of the clinically most important of these are the Class A penicillinases, the Extended-Spectrum β-Lactamases (ESBLs) (TEM, SHV, VEB, or CTX-M the most spread), the AmpC cephalosporinases (usually CMY, FOX, DHA, ACT, or MOX), and the carbapenem-hydrolyzing enzymes in both the serine (i.e., KPC, IMI, SME, or GES) and metallo-enzyme groups (mainly NDM, VIM, or IMP) (Bonomo, 2017). Because of the versatility of these enzymes to evolve as new β-lactams are used therapeutically, combined approaches to antimicrobial therapy may be required (i.e., carbapenems plus colistin). These genes, frequently located on mobile genetic elements, often coexist with other resistance determinants working on other classes of antibiotics such as aminoglycosides and fluoroquinolones (Marti et al., 2014).

A priority to be currently aware and vigilant of is the worldwide recently emerged plasmid-mediated resistance to colistin, another “last-resort” antibiotic used for the treatment of critical infections caused by MDR Gram-negative pathogens (Olaitan et al., 2016).

The Oltrepò Pavese Plan is located in the province of Pavia with an area of ~1,097 km^2^ and a population of 146,579 inhabitants; its name comes to the peculiarity of being south of the river Po, in the full northern Apennines. The Oltrepò Pavese area supports many agricultural and industrial activities causing pollution in the shallower aquifers; for this reason, it represents a good model to evaluate the role of aquatic environment as a potential reservoir for spread and evolution of ARGs and their vectors. The routes by which humans may come in contact with these bacteria include the consumption of crops grown by contaminated sludge used as fertilizer, and/or drinking of water drawn from contaminated ground or surface water. When these resistant bacteria enter humans, they have the opportunity to spread their ARGs to the human microbiome (Wellington et al., 2013).

Only few European studies on surface waters are available; furthermore, due to the lack of local and/or large-scale epidemiological studies, our knowledge on the environmental antibiotic-resistance reservoirs in Italy is still very poor (Perilli et al., 2013; Zanotto et al., 2016).

The purpose of this study was (i) to investigate the occurrence of cefotaxime resistant *Enterobacteriaceae* in Oltrepò Pavese water ecosystem (ii) to determine Gram-negative bacterial counts and percentage of 3GCs resistance, during all the study period, (iii) to characterize the underlying determinants and to assess the clonal features of the most alarming isolates.

## Materials and methods

### Design study and sample collection

The aim of the present 1-year study was to monitor the Gram-negative Oltrepò Pavese area bacterial counts during different months per sampling site, and the corresponding 3GCs resistance percentages. Water samples from wells, streams, and WWTPs of the Oltrepò Pavese area were evaluated for the presence of ESBLs/AmpC, carbapenemases, and *mcr-1* genes in *Enterobacteriaceae*.

The sampling sites are located at south of Po river in the Italian Northern Apennines, in the zone between Voghera, Stradella, and Staffora Valley near Varzi.

This area is characterized by the presence of a sandy-gravelly alluvial deposits covering the marine sediments, which are essentially impermeable (Pilla et al., 2007). Local well have a recharging time of 6 months on average and their structure does not provide a complete isolation from the surface water.

Sampling was carried out once a month—during the period December 2014–November 2015. In the study period, a total of 246 water samples were collected. Each of the single stream sampling site (1T−11T) was visited from 9 up to 11 times, while the 11 wells (1P−13P) were sampled from 10 up to 12 times. The four WWTPs included in the study were less frequently inspected (from three to six times), due to the need for a special authorization to get samples.

**Figure 1:**
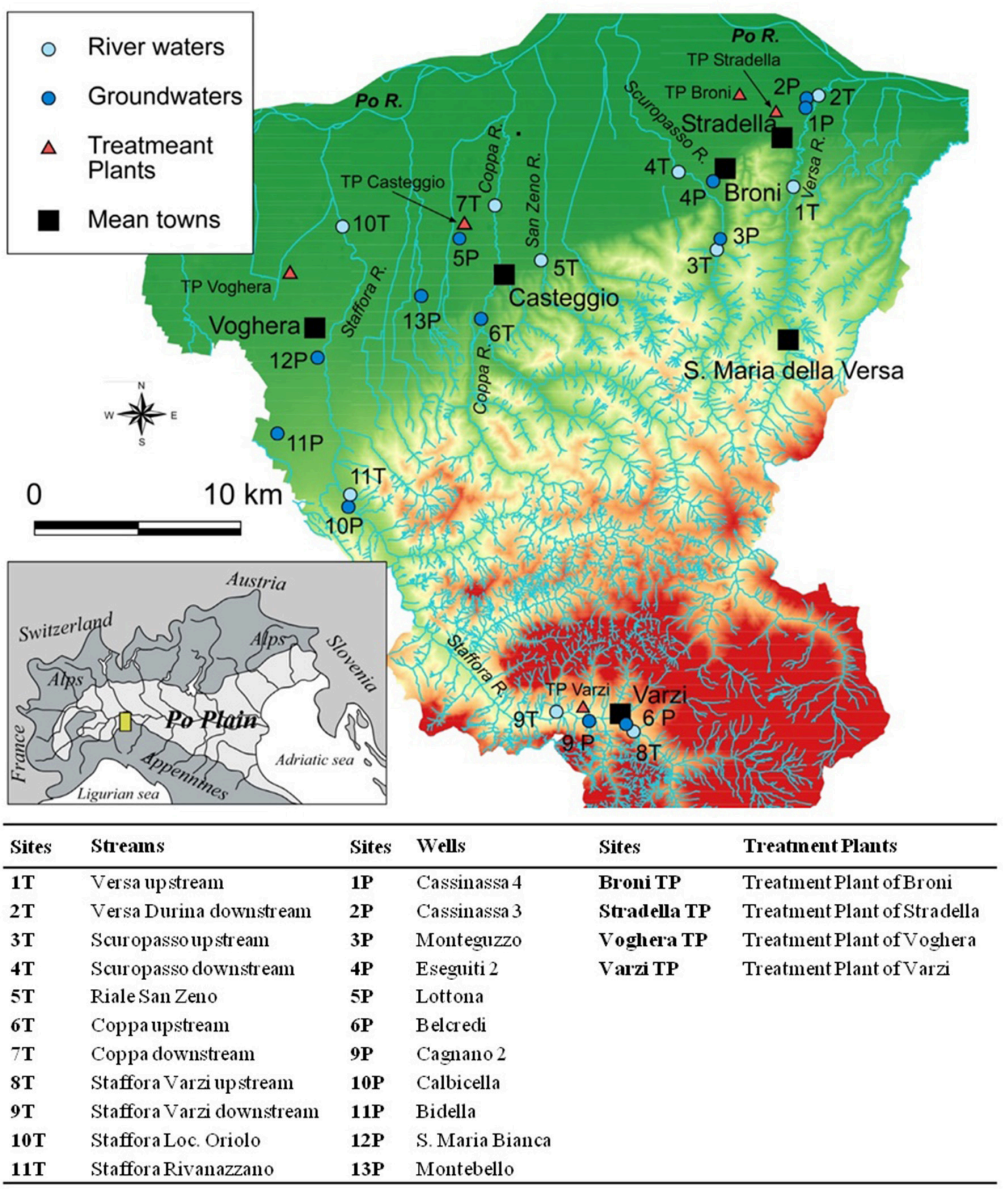
Map of the Oltrepò Pavese Plain showing the sampling sites. P, Well; T, Stream; TP, Wastewater Treatment Plant.

All the sampled wells are part of the Po Plan Public-Supply System, serving freshwater for human consumption in the area. The outflow of four selected WWTPs, located upstream rivers of the same area, was screened starting from April 2015 only (Figure 1).

All water samples were collected in aseptic plastic bottles, stored at 4°C, and analyzed within 24 h after harvesting.

The stream sampling sites have been chosen with different location, upstream, and downstream urban centers, including factories, hospitals, and Long Term Care Facilities (LTCFs).

### Filtration method

A water volume of 100 ml from wells and 1 ml from up/down streams or at the outflow of treatment plants were passed through 0.45 μm-pore size membranes by the filtration method. Filter membranes were placed on Plate Count Agar (PCA), MacConkey Agar (MCA), and selective MCA containing 8 mg/l of CTX (MCA+CTX), to select the potential ESBL/AmpC producers. The bacterial count was estimated after 24 h incubation of the plates at 37°C.

The 3GCs resistance percentage (% R) was calculated by directly comparing the colonies count on the antibiotic plate (MCA+CTX) with the corresponding count on the control plate (MCA) (Watkinson et al., 2007).

### Bacterial identification and susceptibility testing

Each colony grown on selective MCA+CTX and distinguishable for the unique morphotype, was identified at species level. Species identification and susceptibility testing were performed using the semi-automated system MicroScan autoSCAN-4 (Beckman Coulter). Colistin (CO) MIC was confirmed by broth microdilution method. Susceptibility results were interpreted according to the EUCAST 2015 (http://www.eucast.org) clinical guidelines. *Escherichia coli* ATCC 25922 was used as control strain.

Phenotypic ESBL detection was performed by the use of chromogenic media ChromArt ESBL (Biolife Italiana S.r.l., Milano, Italy) and the Double-Disc Synergy (DD) test (Jarlier et al., 1988), using piperacillin-tazobactam, CTX, cefepime, ceftazidime, and aztreonam. A phenotype of both cefoxitin and 3GCs resistance without synergistic activity after DD, was regarded as AmpC-production marker. The strains were phenotypically confirmed for the carbapenemases production by the KPC/MBL Confirm kit (Rosco Diagnostic).

### Molecular characterization of resistance genes

The genomic DNA was extracted using NucleoSpin Tissue (Macherey-Nagel) kit. PCR was used to detect the presence of ESBLs, carbapenemases-, and MCR-1-encoding genes using primers and the annealing temperatures described in Table 1 (Rasheed et al., 1997; Yigit et al., 2001; Perilli et al., 2002; Pagani et al., 2003; Eckert et al., 2004; Liu et al., 2016). In the case of ESBLs and carbapenemases, the above annealing temperatures were always preceded by an initial 95°C × 5 min denaturation step. The 35 cycling conditions comprised denaturation and extension steps at 94°C × 3 min and 72°C × 1 min respectively; the final extension was at 72°C 5 min. *mcr-1* PCR approach was performed using an initial 94°C × 5 min denaturation step; the 35 cycling conditions comprised the denaturation and extension steps at 94°C and 72°C × 30 s, respectively; the final extension was at 72°C 5 min. PCR products were purified using the quantum PrepPCR Kleen Spin Columns kit (ThermoFisher Scientific) and subjected to double-strand sequencing using the automatic sequencer ABI PRISM 3100 genetic analyzer DNA Sequencer (Applied Biosystems, Foster City, CA, USA) and the BigDye Terminator v1.1 Cycle Sequencing kit (Applied Biosystems, Foster City, CA, USA).

**Table 1:** Oligonucleotides used for PCR and sequencing.

**Gene**	**Primer sequences**	**T*a*^a^ (°C)**	**Fragment size (bp)**
*bla*_CTX−M_-type^*^	FW 5′-ATGTGCAGYACCAGTAARGT-3′	50	593
	REV 5′-TGGGTRAARTARGTSACCAGA-3′		
*bla*_CTX−M−1_- group	FW 5′-GGTTAAAAAATCACTGCGTC−3′	50	1,000
	REV 5′- TTGGTGACGATTTTAGCCGC-3′		
*bla*_CTX−M−9_- group	FW 5′- ATGGTGACAAAGAGAGAGTGCA-3′	56	835
	REV 5′- CCCTTCGGCGATGATTCTC-3′		
*bla*_SHV_-type	FW 5′-GCCCGGGTTATTCTTATTTGTCGC-3′	60	900
	REV 5′-TCTTTCCGATGCCGCCGCCAGTCA-3′		
*bla*_TEM_-type	FW 5′-ATGAGTATTCAACATTTCCG-3′	59	800
	REV 5′-CTGACAGTTACCAATGCTTA-3′		
*bla*_KPC_-type	FW 5′-TGTCACTGTATCGCCGTC-3′	55	1,000
	REV 5′-CTCAGTGCTCTACAGAAAACC-3′		
*bla*_VIM_-type	FW 5′-CAGATTGCCGATGGTGTTTGG-3′	55	523
	REV 5′-AGGTGGGCC ATTCAGCCAGA-3′		
*bla*_IMP_*-*type	FW 5′-GGAATAGAGTGGCTTAATTCTC-3′	50	361
	REW 5′-GTGATGCGTCYCCAAYTTCACT-3′		
*bla*_NDM_*-*type	FW 5′-GGTTTGGCGATCTGGTTTTC-3′	52	621
	REW 5′-CGGAATGGCTCATCACGATC-3′		
*bla*_OXA−48_	FW: 5′-TTGGTGGCATCGATTATCGG-3′	52	743
	REV: 5′-GAGCACTTCTTTTGTG ATGGC-3′		
*mcr*-1^*^	FW: 5′-CGGTCAGTCCGTTTGTTC-3′	46	309
	REV: 5′-CTTGGTCGGTCTGTAGGG-3′		

a Ta, annealing temperature;

* *primers used only in the PCR reaction*.

The sequences were analyzed according to the BLAST software program (http://blast.ncbi.nlm.nih.gov/Blast.cgi).

In the case of *Y. enterocolitica* Check-MDR CT103 XL array (Check points Health B.V., Wageningen, The Netherlands) has been used to investigate the *bla* genes content.

### Conjugation transfer experiments and plasmid characterization

Conjugation transfer of resistance determinants was performed in liquid medium using the *E. coli* K12 strain J62 (F^−^, *pro, his, trp, lac*, Sm^r^) and J53 (F^−^, *met, pro*, Rif^r^) as recipients. To evaluate the transferability of CTX- or CO- resistance, the *E. coli* transconjugants were screened using MCA plates supplemented with streptomycin (1,000 mg/l) or rifampicin (100 mg/l) plus CTX (8 mg/l) or CO (2 mg/l), respectively. Azide-resistant *E. coli* J53Az^R^ strain was used as a recipient for carbapenem-resistant *Klebsiella pneumoniae* isolates. MCA supplemented with sodium azide (200 mg/l) and ertapenem (0.5 mg/l) was used to select for transconjugants. Conjugation frequency per recipient was expressed by dividing the number of transconjugants by the initial number of recipients.

Plasmids were typed according to their incompatibility group using the PCR based replicon typing scheme PBRT 2.0 Kit (Diatheva), as described previously (Carattoli, 2009).

### Molecular typing

PFGE was performed on all ESBL-, carbapenemase-, and MCR- positive *E. coli* and *K. pneumoniae* isolates. All the obtained pulsotypes were than compared with the hyper-epidemic clones previously collected in the same area (e.g., 20LOM *E. coli*), Genomic DNA of isolates was analyzed after digestion with *XbaI* restriction enzyme. Fragments were separated on a CHEF DRII system (Bio-Rad, Hercules, CA) at 14°C at 6 V/cm for 20 h with an initial pulse time of 0.5 s and a final pulse time of 30 s. Lambda 48.5 kb concatamers (New England BioLabs, Beverly, MA, USA) were used as molecular size markers.

Dendrograms of strain relatedness were created with Fingerprinting II version 3.0 software (Bio-Rad) using UPGMA. The Dice correlation coefficient was used with a 1.0% position tolerance to analyze the similarities of the banding patterns. The restriction patterns of the genomic DNA from the isolates were analyzed and interpreted according to the criteria described previously (Tenover et al., 1995).

Bacterial strains were cultivated in MCA; one single colony per strain was used for DNA extraction using NucleoSpin Tissue (Macherey-Nagel) kit. MLSTs genes were sequenced using the automatic sequencer ABI PRISM 3100 genetic analyzer DNA Sequencer (Applied Biosystems, Foster City, CA, USA) and the BigDye Terminator v1.1 Cycle Sequencing kit (Applied Biosystems, Foster City, CA, USA).

MLST for *E. coli* was done according to the MLST Databases at University of Warwick (http://mlst.warwick.ac.uk/mlst/dbs/Ecoli) and for *K. pneumoniae* according to the Institute Pasteur MLST (http://bigsdb.pasteur.fr/klebsiella/klebsiella.html). MLST of *Enterobacter cloacae* was done according to the *E. cloacae* MLST website (https://pubmlst.org/ecloacae/) developed by Keith Jolley and sited at the University of Oxford.

### Plasmid characterization

7T*E.coli* plasmid sequencing was done by Illumina MiSeq technology, using Nextera XT kits for library preparation. Reads were assembled using Spades 3.8 (http://cab.spbu.ru/software/spades/).

The detection of resistance genes and plasmid replication sites have been performed on J53R7T*E. coli* using the ResFinder and PlasmidFinder tools in the DTU database (http://www.genomicepidemiology.org/).

Contigs containing plasmid sequences were detected, analyzed, and closed using the software Bandage (https://rrwick.github.io/Bandage/). Open Reading Frames (ORFs) and the relative amino acids were predicted using Artemis (http://www.sanger.ac.uk/science/tools/artemis). The annotation was performed manually using the online blast tool on the ncbi database (https://blast.ncbi.nlm.nih.gov/Blast.cgi?CMD=Web&PAGE_TYPE=BlastHomeNew).

Genbank files were formatted and uploaded using the Sequin software (https://www.ncbi.nlm.nih.gov/Sequin/).

### Statistical analysis

Data were expressed as means (± Standard Error). The difference between means of the bacterial count was tested by the Analysis of Variance (ANOVA). A *P* value below 0.05 was deemed significant.

## Results

### Identification of gram-negative bacteria isolates

A total of 246 water samples was collected; 48% (118/246) from five streams, 43.9% (108/246) were from 11 wells, and 8.1% (20/246) from four WWTPs.

Two hundred and sixty-four non-duplicate microorganisms, one-half (50%; *n* = 132) belonging to enterobacterial species, were overall obtained on MCA+CTX/ChromArt ESBL. The remaining isolates included *Pseudomonas* spp. (19%; *n* = 49), *Acinetobacter* spp. (11%; *n* = 29), *Vibrio fluvialis* (7%; *n* = 18), *Aeromonas hydrophila* (9%; *n* = 25), and other Gram-negative bacteria (4%; *n* = 11).

### Bacterial counts and percentage of 3GCs resistance

The number in average of CFU ml^−1^ of bacteria identified after membrane filtration on PCA ranged from zero to 10,000 (data not shown). High bacterial densities were always observed in the case of WWTPs (5,100–10,000 CFU ml^−1^), while a month to month fluctuating trend have been observed in streams samples, with number in average bacterial counts ranging between 1,023 and 8,272 CFU ml^−1^(Table 2).

**Table 2:** Bacterial counts and percentage of 3GCs resistance for each sampling site.

**Site^a^**		**Avg no. of**		
	**N^b^**	**CFU/ml^−1^ (±SE)**	**% CTX-R^c^**	**% CTX-R range^d^**
1T	11	4, 625 ± 1, 552	41	0–100
2T	11	4, 627.3 ± 1, 551	46	0–100
3T	11	3, 694.9 ± 1, 507	5.3	0–40
4T	11	5, 764.3 ± 1, 997	68	0–100
5T	11	7, 317.3 ± 1, 385	49.5	0–100
6T	9	4, 500 ± 1, 739	42.7	0–100
7T	11	8, 272.7 ± 1, 159	46	0–100
8T	10	1, 023.3 ± 997.4	3.75	0–32.4
9T	11	3, 709.1 ± 1, 504	25.3	0–100
10T	11	3, 696.8 ± 1, 507	6.4	0–40
11T	11	2, 812.7 ± 1, 392	18.8	0–90
1P	12	16.4 ± 6.8	20	0–100
2P	10	2, 033.5 ± 1, 328	12.9	0–100
3P	10	1, 039.5 ± 995	10	0–0.2
4P	10	36 ± 15	10.3	0–77
5P	10	12.9 ± 5.3	10	0–100
6P	10	8, 020 ± 1, 320	7	0–25
9P	10	9, 005 ± 995	13	0–50
10P	11	6, 050 ± 1, 612	6.6	0–25
11P	10	17.5 ± 10	5	0–50
12P	11	1, 003 ± 999	0	0
13P	10	1, 028.2 ± 864	18	0–100
TP Varzi	6	10,000	50.8	25–100
TP Broni	3	10,000	100	100
TP Voghera	3	5, 100 ± 4, 000	75	25–100
TP Stradella	3	6, 733.3 ± 3266	52	25–100

*% CTX-R, % Cefotaxime-resistance; SE, standard error*.

a P, Well; T, Stream; TP, Wastewater Treatment Plant;

b Refers to the number of visits to each site;

c *% CTX-R, percentage of 3GCs- resistant Gram-negatives obtained as an average value, in dependence from the overall visits performed at the same sampling site*.

d *% CTX-R range, percentage 3GCs-resistant Gram-negatives, obtained comparing the bacterial growth on MCA and MCA+CTX, range 0–100%, depending on the single visit and sampling site*.

Concerning the wells results, the bacterial detection frequencies varied between 12.9 and 9,005 CFU ml^−1^; highest densities being found for the three wells named 6P, 9P, and 10P (Table 2).

The percentages of 3GCs-resistant Gram-negatives, obtained comparing the bacterial growth on MCA and MCA+CTX, ranged between 0 and 100%, depending on the single visit and sampling site (Table 2).

### Statistical analysis

Seasonal changes of total bacterial count were statistically evaluated. No significant seasonal effects on total vital count were found analyzing samples from wells and WWTPs.

The lowest bacterial count (as average value) was registered from the streams in October (126.4 ± 34.1). A significant increase (*p* < 0.05) in the streams mean bacterial count was observed, starting from the beginning of winter toward the end of spring. If the highest value of the mean bacterial count (10,000 ± 202.5) has been recorded in April, a significant decrease (*p* < 0.05) was observed from the beginning of summer to the end of autumn.

The highest percentage of CTX-resistant Gram-negative microorganisms (65.65 ± 13.1) was recorded in May. A significant decrease (*p* < 0.05) in the same percentage was observed since the beginning of summer toward the end of autumn. The lowest value was found in September (10.2 ± 7.8). The mean values of both total bacterial count and percentage of CTX-resistant Gram-negatives are shown in Table 3.

**Table 3:** Mean values of total bacterial count and percentage of 3GCs-resistance (±SE) from stream samples during different seasons.

	**Winter**	**Spring**	**Summer**	**Autumn**
Bacterial Count (CFU/ml)	5, 196.485 ± 862	6, 687.636 ± 815.45	3, 137.4 ± 1, 005	2, 231.179 ± 766.8
% CTX-Resistance	36.3 ± 6.9	46.3 ± 7.8	12.8 ± 2.95	15.5 ± 6.2

### Phenotypic testing and antibiotic resistance patterns

A total of 132 enterobacteria grew on MCA+CTX: 82 (62.1%) were from streams, 41 (31.1%) from treatment plants, and 9 (6.8%) from wells samples. Only 30/132 (22.7%) *Enterobacteriaceae* showed a synergic effect by DD phenotypic test using 3GCs and piperacillin-tazobactam; 15/30 were *E. coli*, 7/30 *Yersinia enterocolitica*, 5/30 *K. pneumoniae*, 2/30 *Klebsiella oxytoca*, and 1/30 *E. cloacae*. Two (1.5%) out of 132 *Enterobacteriaceae*, were phenotypically confirmed as carbapenemases producers by KPC/MBL kit, while 1/132 (0.8%) was identified as AmpC-producer.

All the collected *Y. enterocolitica* strains showed clinical resistance to 3GCs; the 12.5% were also chloramphenicol and fosfomycin resistant. All the *E. coli* and *Klebsiella* spp. isolates showed resistance to 3GCs; the 13.3% (*n* = 2) of *E. coli* and 22.2% (*n* = 2) of *Klebsiella* spp. resulted, in addition, resistant to the three main carbapenems (ertapenem, meropenem, imipenem). Notably, 60% (*n* = 9) ESBLs-producing *E. coli* isolates showed fluoroquinolones resistance (Table 4).

**Table 4:** Molecular characteristics of ESBLs/AmpC/carbapenemases-producing isolates recovered from streams, wells, and WWTPs.

**MLST (ST)**	**Isolate**	**Date of Isolation (Month, Year)**	**Origin^a^ (Sample)**	**Resistances^b^**	**Resistance determinant**	**Inc(Replicon)^c^**
993	6P *E. coli*	June 2015	6P	AMC, AMP, CTX, ERT	CTX-M-1	IncP (P) , IncL (L), IncF (FIA)
NTe	7T *E. cloacae*	March 2015	7T	AMC, AMP, CAZ, PIP, CTX	CTX-M-14	IncF (FIA), IncU (U)
131	1T *E. coli*	January 2015	1T	AMP, CAZ, CTX, FEP, CIP, PIP, LEV, MOXI, NOR, TOB, TMS	CTX-M-1	IncF (FIB), Inc FII (FII)
94	3T *E. coli*	January 2015	3T	AMC, AMP, CAZ, CTX, FEP, PIP, ERT, MEM, TZP, CIP, CL, MOXI, NOR	CTX-M-28, TEM-1	IncP (P), IncF (FIB)
6151	11T *E. coli*	May 2015	11T	AMC, AMP, CAZ, CTX, FEP, PIP, TZP	SHV-12	IncN (N), Inc B/O (B/O), IncF (FIB)
5717	1TP *E. coli*	June 2015	TP Broni	AMC, AMP, CTX, CAZ, FEP, PIP, CIP, LEV, MOXI, NOR, TOB, CL, FOS	CTX-M-28	IncHI2 (HI2), Inc B/O (B/O), IncF (FIA, FIB), IncA/C (A/C), IncFII (FII)
5590	2TP *E. coli*	July 2015	TP Broni	AMP, FEP, CTX, CAZ, PIP, CIP, CL, MOXI, NOR, LEV,	CTX-M-14	IncX1 (X1)
7519	3TP *E. coli*	October 2015	TP Broni	AMP, FEP, CTX, CAZ, PIPCIP, CO, MOXI, NOR, LEV, TMS, TOB	CTX-M-138	IncHI2 (HI2), IncM (M)
5080	1TP *E. coli*	June 2015	TP Stradella	AMP, CTX, FEP CIP, CO, GM, MOXI, NOR, LEV, TMS, TOB	CTX-M-1	IncF (FIA, FIB)
3132	1TP *E. coli*	May 2015	TP Varzi	AMP, CTX, FEP, PIP, TMS	CTX-M-15	IncF (FIA), IncT (T)
399	2TP *E. coli*	May 2015	TP Varzi	AMP, CTX, CIP, LEV, MOXI, NOR, PI, TMS	CTX-M-15	IncF (FIA)
7228	3TP *E. coli*	May 2015	TP Varzi	AMP, CTX, CAZ, FEP, PIP	CTX-M-138	IncN (N), IncF (FIA, FIB)
2432	4TP *E. coli*	June 2015	TP Varzi	AMP, CTX, FEP, PIP CIP, MOXI, NOR, LEV	CTX-M-1	IncM (M), IncN (N), HIBM (NA)d, FIBM (NA)d
2208	5TP *E. coli*	June 2015	TP Varzi	CIP, MOXI, NOR, LEV	CTX-M-14	IncL (L), IncF (FIA, FIB)
362	6TP *E. coli*	July 2015	TP Varzi	AMP, CTX, CAZ, PIP, CIP, MOXI, NOR, LEV, GM, TMS	CTX-M-1, SHV-5	IncP (P), IncF (FIA, FIB)
10	7T *E. coli*	November 2015	7T	AMC, AMP, CAZ, PIP, TMS, CO	CTX-M-1, SHV-12, MCR-1.2	IncX4 (X4), IncX3 (X3)
NTe	6T *K. oxytoca*	April 2015	6T	AMC, AMP, CAZ, PIP, CTX	SHV-12	IncHI2 (HI2), IncN (N), Inc F (FIA)
NTe	9T *K. oxytoca*	December 2014	9T	PIP, AMP, CTX, FEP, FOS	CTX-M-1	IncN (N), R (NA)d
258	9P *K. pneumoniae*	June 2015	9P	AMC, AMP, CAZ, CTX, PIP, CO	KPC-3	IncFII K (FIIk, FIB KQ)
456	2T *K. pneumoniae*	January 2015	2T	AMC, AMP, CAZ, CTX, FEP, PIP, CL, GM	CTX-M-1	IncN (N)
1266	1TP *K. pneumoniae*	May 2015	TP Varzi	AMP, CTX, FEP, PIP	CTX-M-15	IncF (FIA), IncT (T)
1601	2TP *K. pneumoniae*	May 2015	TP Varzi	AMC, AMP, CAZ, FEP, PIP, CL	CTX-M-28	IncHI1 (HI1), Inc B/O (B/O), IncF (FIA)
194	3TP *K. pneumoniae*	May 2015	TP Varzi	AMP, CAZ, CTX, FEP, PIP, TMS	CTX-M-15, TEM-1	IncM (M), IncN (N), IncB/O (B/O), IncF (FIIK)
1601	4TP *K. pneumoniae*	May 2015	TP Varzi	AMP, CTX, CAZ, FEP, PIP, CL, CIP, MOXI, NOR, LEV	CTX-M-15	IncHI1 (HI1), IncL (L)
307	5TP *K. pneumoniae*	July 2015	TP Varzi	AMC, AMP, CTX, CAZ, FEP, MEM, ERT, TZP, CIP, NOR, MOXI, FOS, GM, TMS, CL	TEM-1, KPC-2	IncFIIK (FIIk, FIB KN, FIB KQ)
NTe	1T *Y*. *enterocolitica*	June 2015	1T	AMC, AMP, CTX, FOS	Other mechanism	NTe
NTe	2T *Y. enterocolitica*	March 2015	2T	AMC, AMP, CTX, PIP	Other mechanism	NTe
NTe	3T *Y. enterocolitica*	May 2015	3T	AMC, AMP, CTX, PIP	Other mechanism	NTe
NTe	6T *Y. enterocolitica*	January 2015	6T	AMC, AMP, CTX, CAZ, CL	Other mechanism	NTe
NTe	6T *Y. enterocolitica*	February 2015	6T	AMC, AMP, CTX, CAZ	Other mechanism	NTe
NTe	6T *Y. enterocolitica*	March 2015	6T	AMC, AMP, CTX, CAZ	Other mechanism	NTe
NTe	11T *Y. enterocolitica*	May 2015	11T	AMC, AMP, CTX, PIP	Other mechanism	NTe
NTe	11T *Y. enterocolitica*	June 2015	11T	AMC, AMP, CTX, PIP	DHA-type	NTe

a P, well; T, stream; TP, Treatment Plant;

b *AMC, amoxicillin/clavulanic acid; AMP, ampicillin; CAZ, ceftazidime; CIP, ciprofloxacin; CL, chloramphenicol; CO, colistin; CTX, cefotaxime; ERT, ertapenem; FEP, cefepime; FOS, fosfomycin; GM, gentamicin; LEV, levofloxacin; MEM, meropenem; MOXI, moxifloxacin; NOR, norfloxacin; PIP, piperacillin; TZP, piperacillin-tazobactam, TOB, tobramycin; TMS, trimetoprim/sulfametoxazol*.

c Inc, incompatibility group;

*^d^NA, not assigned; ^e^NT, not tested*.

Only 3/15 (20%) of the *E. coli* (7T*E. coli*, 1TP*E. coli* Stradella, 3TP*E. coli* Broni) and 1/7 (14.2%) of the *K. pneumoniae* (9P) out of the 33 ESBLs (*n* = 30)/AmpC (*n* = 1)/carbapenemases (*n* = 2) phenotypically suspected enterobacteria, resulted CO-resistant (MIC > 4 mg/l) by MicroScan-A4 System.

Clinical CO resistance was confirmed only for the strain named 7T*E. coli* (MIC = 8 mg/l) by MIC broth microdilution method.

KPC/MBL confirm kit test showed a positive result for the 9P and 5TP*K. pneumoniae* strains, obtained on June 2015 and July 2015 from a well and a WWTP in the Varzi area, respectively. The boronic acid synergistic effect suggested the production of a KPC-type enzyme.

### Detection and characterization of resistance genes

*A bla*_CTX−M_-type gene, was identified in a total of 21/33 (63.6%) isolates (*n* = 14 *E. coli*; *n* = 5 *K. pneumoniae*; *n* = 1 *K. oxytoca; n* = 1 *E. cloacae*) from all water compartments (Table 4).

In four cases (3T*E. coli*; 3TP*K. pneumoniae*; 7T *E. coli*; 6TP *E. coli* Varzi) the *bla*_CTX−M_-type gene co-existed with other β-lactamase determinants, as shown in Table 4. The most identified *bla*_CTX−M_ variant was *bla*_CTX−M−1_ (8/21; 38%), followed by the *bla*_CTX−M−15_ (5/21; 23.8%), the *bla*_CTX−M−14_ and *bla*_CTX−M−28_ (3/21, each; 14.3%, each), and *bla*_CTX−M−138_ (2/21; 9.5%).

A *bla*_SHV−12_ gene was present in three isolates, named 11T*E. coli*, 7T*E. coli*, and 6T*K. oxytoca*.

Only 1/8 Chromogenic ESBL-agar selected *Y. enterocolitica* strains (the cefoxitin resistant 11T) harbored a *bla*_DHA_-type gene, while the remaining seven resulted negative for any *bla* resistance gene by Check-MDR CT103 XL array.

PCR and sequencing confirmed the presence of the *bla*_KPC−2_ and *bla*_KPC−3_ determinants in the 5TP and 9P*K. pneumoniae* strains, respectively. A co-occurrence of *mcr-1.2, bla*_CTX−M−1_, and *bla*_SHV−12_ genes was detected by *in-silico* plasmid characterization in the 7T*E. coli* collected on November 2015 from Coppa stream (Table 4).

### Conjugation experiments results

Conjugation experiments were performed on all the 25 ESBLs/KPC positive strains: 15 *E. coli*, seven *K. pneumoniae*, two *K. oxytoca*, one *E. cloacae*.

Lateral transfer of resistance genes was observed in 15/25 (60%) isolates: 11 *E. coli*, three *K. pneumoniae*, and one *K. oxytoca*, as shown in Table 5. The resistance profiles of donors and transconjugants confirmed lateral transfer of 3GCs, carbapenems (ertapenem), and colistin resistance. PCR analysis confirmed the presence of the resistance genes in all the transconjugants (Table 5).

**Table 5:** Phenotipical and molecular characteristics of 3GCs-resistant transconjugants; comparison with *E. coli* and *Klebsiella* spp. donors resistance and β-lactamases.

**Isolates**	**Resistance^a^**	**Enzyme**	**Inc (Replicon)^b^**
1T *E. coli*	**AMP**, **CAZ**, **CTX**, **FEP**, **CIP**, **PIP**, **LEV**, **MOXI**, **NOR**, TOB, **TMS**.	**CTX-M-1**	IncF (FIB)
3T *E. coli*	AMC, **AMP**, CAZ, **CTX**, **FEP**, **PIP**, ERT, MEM, TZP, **CIP**, **CL**, **MOXI**, **NOR**.	**CTX-M-28**, **TEM-1**	IncP (P), IncF(FIB)
7T *E. coli*	AMC, AMP, CAZ, PIP, TMS, **CO**.	CTX-M-1, **MCR-1.2**, SHV-12	IncX4 (X4)
11T *E. coli*	**AMC**, AMP, CAZ, **CTX**, FEP, **PIP**, TZP.	**SHV-12**	IncF (FIB)
1TP *E. coli* Broni	AMC, **AMP**, **CTX**, CAZ, **FEP**, **PIP**, **CIP**, **LEV**, **MOXI**, **NOR**, TOB, **CL**, FOS.	**CTX-M-28**	IncHI2 (HI2), IncF (FIA, FIB)
2TP *E. coli* Broni	**AMP**, CAZ, **CTX**, **FEP**, **PIP**, CIP, **LEV**, MOXI, NOR, CL.	**CTX-M-14**	IncX1(X1)
1TP *E. coli* Varzi	**AMP**, **CTX**, **FEP**, **PIP**, TMS.	**CTX-M-15**	IncT (T)
2TP *E. coli* Varzi	**AMP**, **CTX**, **CIP**, **LEV**, **MOXI**, **NOR**, PI, TMS.	**CTX-M-15**	IncF (FIA)
3TP *E. coli* Varzi	**AMP**, **CTX**, CAZ, FEP, PIP.	**CTX-M-15**, TEM-1	IncF (FIA, FIB)
6TP *E. coli* Varzi	**AMP**, **CTX**, **CAZ**, **PIP**, **CIP**, **LEV**, **MOXI**, **NOR**, **TOB**, **TMS**.	**CTX-M-1**, **SHV-5**	IncF (FIA, FIB)
1TP *E. coli* Stradella	**AMP**, **CTX**, **FEP**, CIP, CO, GM, MOXI, NOR, LEV, **TMS**, TOB.	**CTX-M-1**	IncF (FIA, FIB)
1TP *K. pneumoniae* Varzi	**AMP**, **CTX**, **FEP**, **PIP**.	**CTX-M-15**	IncF (FIA)
3TP *K. pneumoniae* Varzi	**AMP**, CAZ, **CTX**, FEP, PIP, TMS.	**CTX-M-28**	IncN (N)
5TP *K. pneumoniae* Varzi	**AMC**, **AMP**, **CTX**, CAZ, FEP, MEM, **ERT**, **TZP**, CIP, NOR, MOXI, FOS, CL, GM, TMS.	**TEM-1**, **KPC-2**	IncFIIk (FIIk, FIBKQ)
9T *K. oxytoca*	**AMP**, **CTX**, **FEP**, FOS, **PIP**.	**CTX-M-1**	IncN (N), R (NA^c^)

a AMC, amoxicillin/clavulanic acid; AMP, ampicillin; CAZ, ceftazidime; CIP, ciprofloxacin; CL, chloramphenicol; CO, colistin; CTX, cefotaxime; ERT, ertapenem; FEP, cefepime; FOS, fosfomycin; GM, gentamicin; LEV, levofloxacin; MEM, meropenem; MOXI, moxifloxacin; NOR, norfloxacin; PIP, piperacillin; TZP, piperacillin-tazobactam; TOB, tobramycin; TMS, trimetoprim/sulfametoxazol;

b *Inc, incompatibility group*,

c *NA, not assigned*.

*The antibiotic resistance and the determinants transferred in the transconjugants are underlined and provided in bold*.

The transfer of CTX resistance was observed at a frequency of ~10^−3^ transconjugants per recipient. Compared to the *E. coli* J53 and J62 strains used as recipients, the transconjugants exhibited a decreased susceptibility to 3GCs (data not shown).

While in the case of KPC-3-producing 9P*K. pneumoniae* the conjugation experiment failed, the transfer of the CO resistance trait from MCR-1.2-producing 7T*E. coli* to J53 (met, pro-, rif^r^) *E. coli* K12 was possible, at a frequency of ~10^−2^ transconjugants per recipient. Susceptibility testing by autoSCAN-4 System revealed a MIC ≥ 4 mg/l for the *mcr-1*-harboring J53R7T *E. coli*.

PCR and sequencing analysis on J53R7T *E. coli* yielded positive results for the presence of the *mcr-1.2* gene. Inc group plasmid analysis performed on J53R7T*E. coli* using PBRT 2.0 kit, showed that *mcr-1.2* resistance determinant was located in plasmid belonging to the IncX4 Group. In addition to the IncX4, also IncX3 incompatibility group was observed in the donor 7T *E. coli*.

### Molecular typing

**Figure 2:**
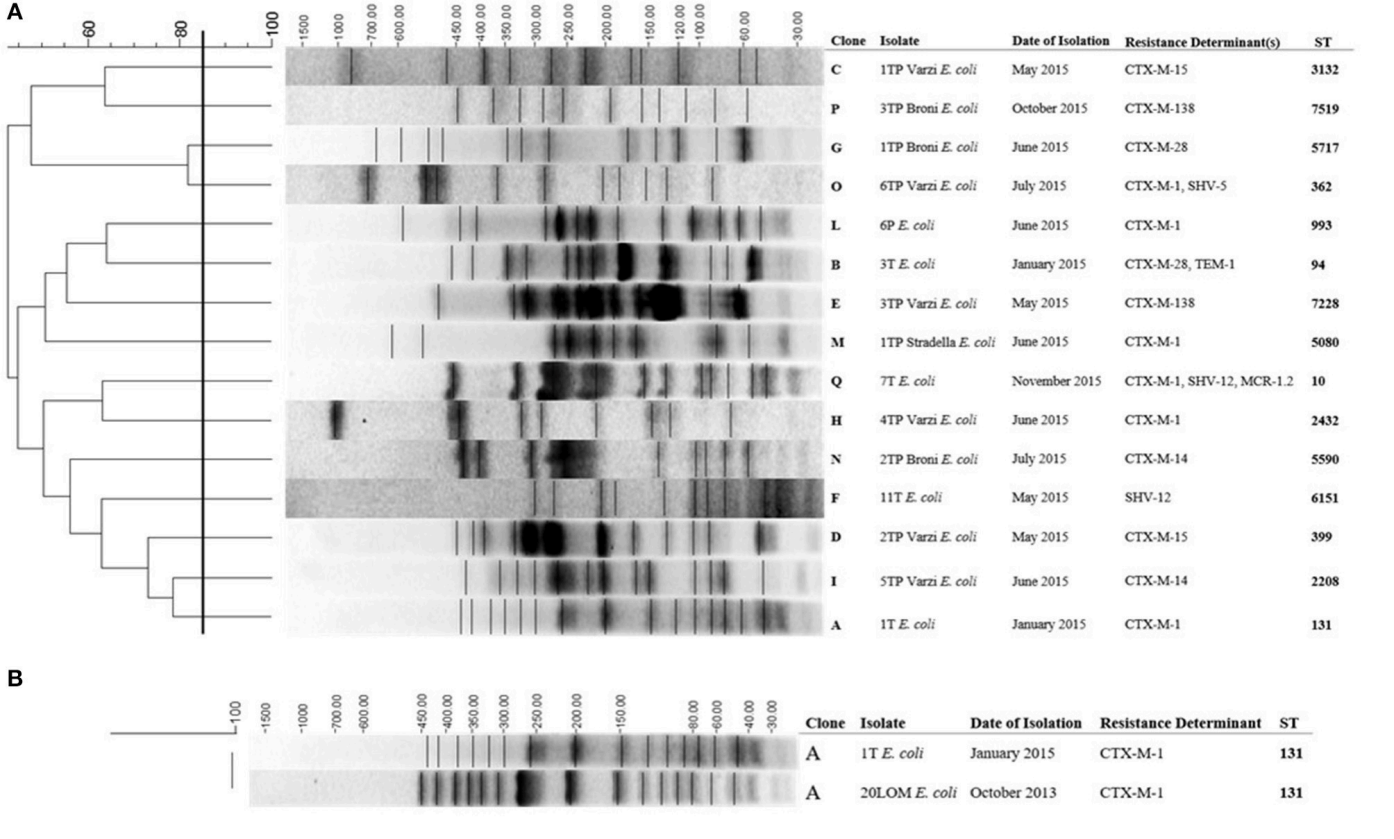
**(A)** Cluster analysis of the ESBL-producing *E. coli* isolates. **(B)** Comparison between the profiles of 1T *E.coli* and 20LOM *E. coli*. Date of isolation (month, year), resistance determinants and ST are also included. The scale bar at the top (left) indicates similarity coefficient (%).

**Figure 3:**
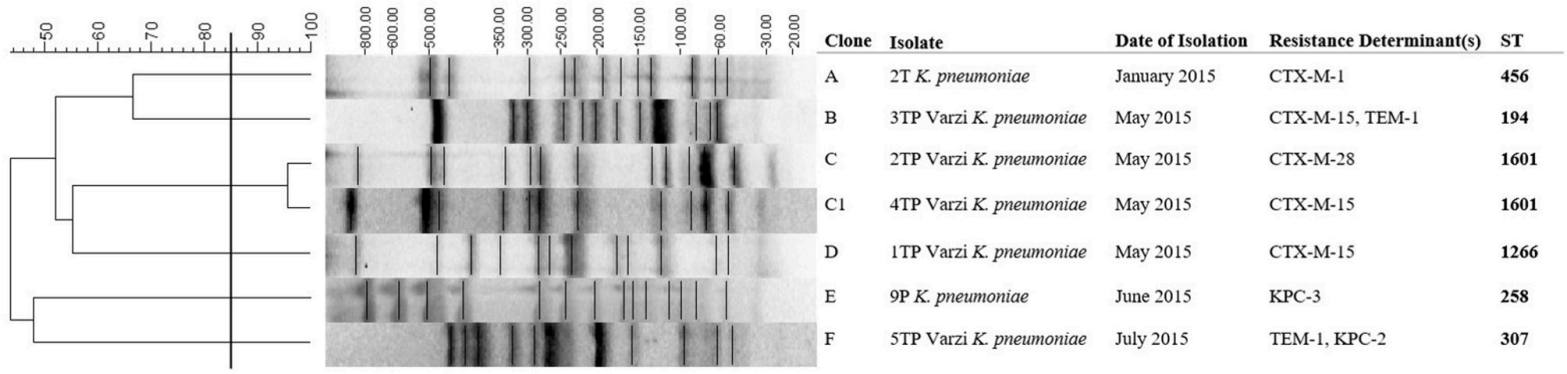
Cluster analysis of the seven ESBLs- or carbapenemases- producing *K. pneumoniae*. Date of isolation (month, year) and resistance determinants are also included. The scale bar at the top (left) indicates similarity coefficient (%).

PFGE analysis carried out on all 15 ESBLs-producing *E. coli* showed clonal diversity (Figure 2). The 1T*E. coli* strain resulted identical to the 20LOM *E. coli* ST 131 (100% similarity between pulsotypes), previously collected in the same area (Figure 2B). Five out of seven ESBLs- and/or carbapenemases- producing *K. pneumoniae* were unique by PFGE (Figure 3). Multiple MLST lineages were identified, coherently with the previously obtained PFGE clonal relationships. Among these, were detected the hyperepidemic clones ST131 *E. coli* and ST258 *K. pneumoniae* (Table 4). The ST1601 was shared by the two PFGE clonally related strains (but showing different resistance profiles and *bla*_CTX−M_ variants) collected in May 2015 from Varzi WWTP.

### Plasmid characterization

**Figure 4:**
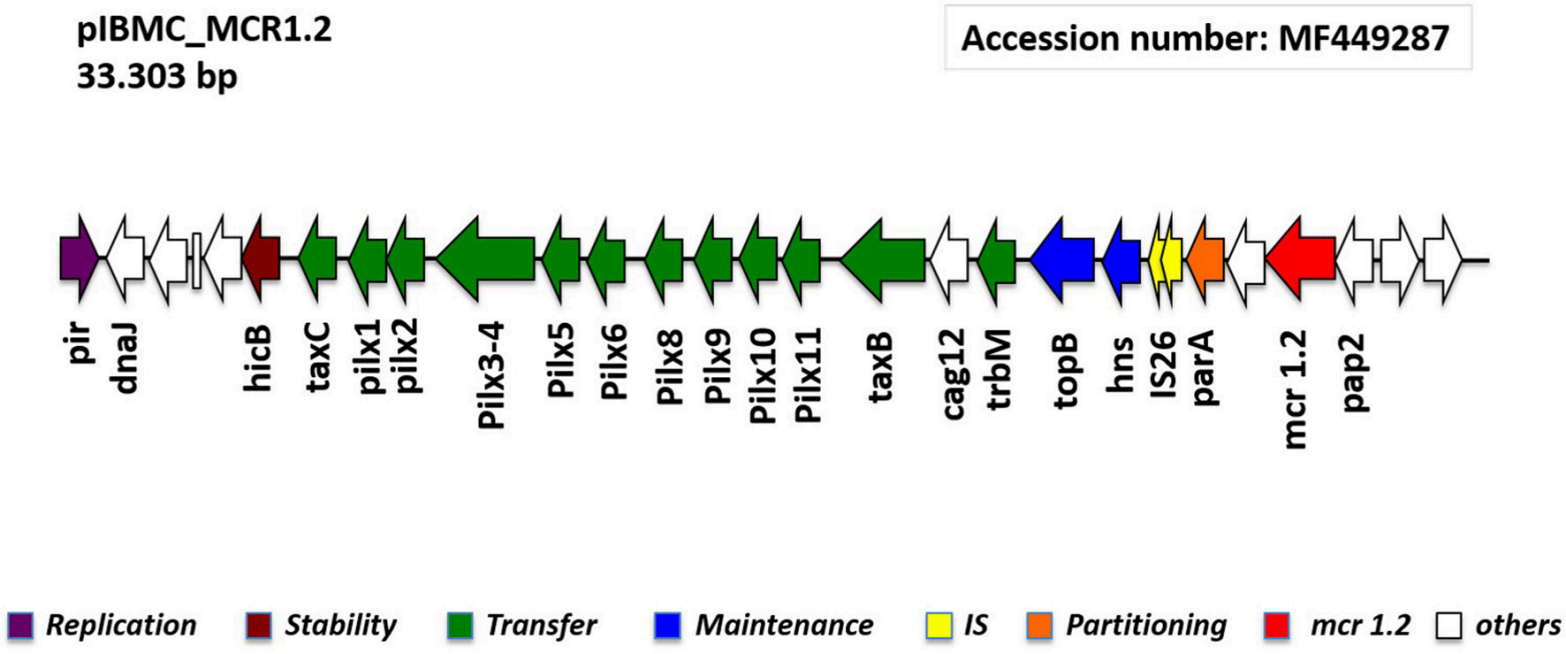
Linear map of the pIBMC_MCR1.2 plasmid, showing the genetic structures surrounding *mcr-1.2* gene.

*In-silico* analysis of the NGS data of the transconjugant J53R7T*E. coli*, revealed the presence of the CO resistance gene *mcr-1.2* on an IncX4 plasmid. Using Bandage, the plasmid was extracted and closed resulting in a 33,303 bp circular plasmid named pIBMC-MCR1.2 (Figure 4).

pIBMC-MCR1.2 showed 99% similarity with the recently reported pMCR1.2-IT plasmid detected in a KPC-3-producing ST512 *K. pneumoniae* clinical isolate collected in Italy (Di Pilato et al., 2016). Other similar IncX4 plasmids carrying *mcr-1* gene were reported in China from a clinical *K. pneumoniae* isolate, in Estonia from a pig sludge *E. coli* isolate, and in South Africa from an *E. coli* clinical isolate (Li et al., 2016; Poirel et al., 2016). In all these plasmids, the *mcr* neighboring regions were highly similar, with the downstream presence of *pap2* gene (encoding for a putative PAP family transmembrane protein), and the absence of the mobilization region IS*Apl1*.

The plasmid pIBMC-MCR1.2 has an average G/C-content of 42%; it comprises 26 ORFs, of which 21 encoding proteins with known functions and five hypothetical proteins. Any additional resistance genes are carried on this plasmid. The plasmid contains a region responsible for mobilization (14.1 kb) and another for replication (1 kb) and maintenance (2.7 kb) (Figure 4).

## Discussion

The occurrence of antibiotic-resistant bacteria from European aquatic sites is increasingly described (Zarfel et al., 2017).

The dissemination in such settings of ESBLs-producing *Enterobacteriaceae* could be particularly worrisome, due to the ability of nosocomial pathogens to transfer ARGs among different hosts and environments (Machado et al., 2009).

This study describes the occurrence of ESBLs, acquired cephalosporinases and carbapenemases among *Enterobacteriaceae* from surface, ground water and WWTP aquatic compartments of the Oltrepò Pavese Plain, Italy. Moreover, this is the first report on the presence of a CTX-M-1, SHV-12 ST10 *E. coli* strain harboring a *mcr-1.2* gene from an Italian stream. CTX-M-type producing ST10 *E. coli* was already reported from farm animals, healthy, and hospitalized humans (Hansen et al., 2014).

The Oltrepò Pavese area is densely populated (355 inhabitants/km^2^ in average), supports many agricultural (as vineyards, orchards), intensive livestock, and industrial activities. Although the study was undertaken in a restricted area, the microbiological results here presented can be regarded as representative, since this hydrogeological site is typical of many other Po plain zones.

During one-year period, a total of 33 CTX-M-, SHV-, DHA-, KPC-type producing *Enterobacteriacae* were identified from 11 wells, five streams, and four WWTPs. A β-lactamase production mechanism was not detected in a large amount of the selected 3GCs resistant enterobacterial species (*n* = 99/132; 75%).

High levels of bacterial contamination and CTX-resistance rates were constantly observed in WWTPs, while seasonal changes—with highest values in spring—were recorded from stream samples. Moreover, fluctuations occurred depending on the sampling sites location, up- or downstream the rivers. The above trend, that well highlights how the local anthropogenic activities directly affect the water quality, was observed also in a recent research from Guadeloupe (Guyomard-Rabenirina et al., 2017).

Surprisingly, high bacterial counts were detected sampling 2/11 wells located nearby the heavily contaminated Varzi WWTP; these data confirm the huge pollution risks existing in this short term (about 60 days) ground water recharge area.

The CTX-M-type ESBLs resulted the most spread 3GCs hydrolyzing enzymes among the collected isolates. The overall β-lactamases detected were mainly of CTX-M-type, followed by SHV-type, TEM-1, and KPC-type.

The high (60%) fluoroquinolones non-susceptibility level detected in 3GCs resistant *E. coli* represents a particular concern, as this antibiotic class is frequently employed in treatment of urinary tract infections.

Fifteen out of 33 (45.4%) β-lactamases-producing *Enterobacteriacae* were collected from WWTPs.

Although in wells 3GCs-resistance rates resulted very low, a ST258 KPC-3-producing 9P*K. pneumoniae* strain has been found in Varzi area. To our knowledge, only one description of an environmental strain ST512 KPC-3-producing *K. pneumoniae* was previously reported in Central Italy from a WWTP (Perilli et al., 2013). In 2011, the European Antimicrobial Resistance Surveillance Network (EARS-Net) described an ongoing worrisome increasing trend of carbapenemase-producing *K. pneumoniae* from invasive samples in Italian Intensive Care Units. Moreover, recent National data show that KPC-producing *K. pneumoniae* Clonal Complex 258 is also frequently found in patients of geriatric or medicine wards (Cristina et al., 2016).

Seven out of eight *Y. enterocolitica*, although initially suspected as ESBLs-positive using phenotypic tests, resulted *bla* genes negative by microarray. The detection of *Y. enterocolitica* showing CTX and cefepime clinical resistance is not common in our country. Thereby, as the intrinsic AmpC production is not enough to explain this phenotype, other mechanisms as porin loss and/or over-expression of efflux systems could be responsible in the resistance detected in our isolates (Frazão et al., 2017).

The plasmid-mediated *mcr-1* gene has recently been reported from food animals, vegetables, asymptomatic carriers, and hospitalized patients in China (Liu et al., 2016). Since then, it has been across five continents (Wang et al., 2017). Regarding the environmental compartment, *mcr-1* was reported in Malaysa (Zurfuh et al., 2016), Switzerland river (Li et al., 2016), and Spain sewage water (Ovejero et al., 2017). In the present study, a *mcr-1.2* variant was detected in the 7TE. *coli* on a conjugative IncX4 plasmid named pIBMC-MCR1.2. Other similar IncX4 plasmids carrying *mcr-1* determinat were reported in China, Estonia, South Africa, and very recently Italy, in different *Enterobacteriaceae* from both clinical and animal origin (Di Pilato et al., 2016; Li et al., 2016; Poirel et al., 2016). In the Italian strain, in particular, the *mcr1.2* gene is carried on a plasmid showing 99% similarity with the here reported pIBMC-MCR1.2.

The spreading potential of *mcr-1*-harboring plasmids in MDR microorganisms poses significant challenges for clinical treatment and infection control strategies, as when β-lactams, aminoglycosides or quinolones are ineffective, colistin serve as the final alternative. The advent of transmissible colistin resistance indicates that the evolution of *Enterobacteriaceae* from extensively to pan-drug resistant is inevitable (Biswas et al., 2012).

The occurrence of ESBLs-, DHA-, KPCs-, MCR-1.2- environmental *Enterobacteriaceae* highlights the importance to improve surveillance and remediation actions on surface and ground waters of Oltrepò Pavese area. In this regard, and out of the aims of this report, we observed clonal relatedness between the 1T*E. coli* and the 20LOM*E.coli* strain isolated in October 2013 from a Long Term and Rehabilitation Facility located near the Versa stream (Figure 2B). Appropriate measures urgently need to be enforced in order to reduce the anthropogenic burden of antibiotic resistance. Improvement of water status is of major concern: new strategies for the treatment of wastewaters, i.e., the use of sand filters or more-stringent chlorine disinfection, need to be taken into consideration to prevent resistant bacteria from being released into the aquatic environment.

The current results support the hypothesis that environmental water might represent an important hidden resistance reservoir.

## Nucleotide sequence accession number

The complete sequence of the pIBMC_MCR1.2 plasmid was deposited into GenBank under the accession number MF449287.

## Author contributions

MC and EN played an important role in interpreting the results and in writing the manuscript. MS and GP helped to acquired data. FZ, SP, FN, VM, AP, IB, and MD carried out experimental work. LP and RM supervised the experiments and revised the final manuscript, which was approved by all authors.

### Conflict of interest statement

The authors declare that the research was conducted in the absence of any commercial or financial relationships that could be construed as a potential conflict of interest.
